# Senex

**Published:** 1872-07

**Authors:** 


### EDITORIAL AND MISCELLANEOUS.

From the present entirely harmonious relations existing among the medical profession of Georgia—to which, as we believe, this journal has largely contributed—we occupy a stand-point which will enable us to make it more valuable and interesting in the future. We have reason to believe that the number of the best men in the State, whose contributions will be found in our columns, will be constantly increasing. We shall be obliged to our friends everywhere for any aid they may be disposed to extend in the way of our circulation, and especially for contributions to our pages. We can honestly say that we have no ill will, envy or jealousy toward any legitimate member of the profession, and desire to do the greatest amount of good to the greatest number.

"Senex."—Over the above signature, we have received from a gentleman of the highest character, personally and professionally, and a friend of many years since, a communication, in connection with which he prefers the "incog." He says, in a private note: "I feel that we old doctors may perhaps be of use in putting brakes to the machine, so that the youngsters don't run it too fast." His article will be read with interest, and will we hope, be followed by others.

